# Impact of Hepatitis Delta Virus Infection on the Selection of Hepatitis B Surface Antigen Mutations

**DOI:** 10.3390/genes15080982

**Published:** 2024-07-25

**Authors:** Kabo Baruti, Wonderful T. Choga, Bonolo B. Phinius, Basetsana Phakedi, Lynnette Bhebhe, Gorata G. A. Mpebe, Patience C. Motshosi, Tsholofelo Ratsoma, Sikhulile Moyo, Mosimanegape Jongman, Motswedi Anderson, Simani Gaseitsiwe

**Affiliations:** 1 Research Laboratory, Botswana Harvard Health Partnership, Gaborone Private Bag BO 320, Botswana; kbaruti@bhp.org.bw (K.B.); wchoga@bhp.org.bw (W.T.C.); bphinius@bhp.org.bw (B.B.P.); bphakedi@bhp.org.bw (B.P.); lbhebhe@bhp.org.bw (L.B.); gmpebe@bhp.org.bw (G.G.A.M.); pmotshosi@bhp.org.bw (P.C.M.); tratsoma@bhp.org.bw (T.R.); smoyo@bhp.org.bw (S.M.); jongmanm@ub.ac.bw (M.J.); manderson@bhp.org.bw (M.A.); 2 Department of Biological Sciences, Faculty of Science, University of Botswana, Gaborone Private Bag 00704, Botswana; 3 School of Allied Health Professions, Faculty of Health Sciences, University of Botswana, Gaborone Private Bag 00704, Botswana; 4 Department of Immunology and Infectious Diseases, Harvard T.H. Chan School of Public Health, Boston, MA 02115, USA; 5 Department of Pathology, Division of Medical Virology, Stellenbosch University, Cape Town 7535, South Africa; 6 Africa Health Research Institute (AHRI), Durban 4013, South Africa; 7 The Francis Crick Institute, London NW1 2BE, UK

**Keywords:** mutations, hepatitis delta virus, hepatitis B virus, people living with HIV, Botswana

## Abstract

The interaction of multiple viruses in one host is thought to enhance the development of mutations. However, the impact of hepatitis D virus (HDV) positivity on the development of unique hepatitis B virus (HBV) mutations among people living with human immunodeficiency virus (HIV) (PLWH) remains poorly understood in African countries, including Botswana. We used HBV sequences generated from the Botswana Combination Prevention Project (BCPP), which is the largest pair-matched cluster-randomized HIV trial in Botswana. Only participants with available HBV sequences (*n* = 55) were included in our study ([HIV/HBV-positive (*n* = 50) and HIV/HBV/HDV-positive (*n* = 5)]. Geno2pheno was used to determine HBV genotypes, and HBV surface region sequences (all subgenotype A1) were aligned in AliView for mutational analysis, while the impact of mutations was assessed using Phyre2. Our results identified 182 common mutations between the two groups. In the HIV/HBV/HDV cohort, only three mutations (*L95W*, *W156Q*, *C221Y*) were classified as deleterious, with only *L95W* being the most frequent. In the HIV/HBV cohort, four mutations (*W199R*, *C221A*, *C221S*, *W223G*) were also classified as deleterious. Our results demonstrate the presence of unique HBV mutations among the HIV/HBV/HDV-positive cohort. Functional characterization of these mutations is recommended to determine their effect on HDV.

## 1. Introduction

Approximately 15–20 million of the 254 million people living with chronic hepatitis B virus (HBV) are infected with hepatitis delta virus (HDV) [1], which translates to a prevalence of approximately 4.5% [2]. HDV is a satellite virus that depends on envelope proteins provided by HBV for successful replication; therefore, the highest burden of HDV is found in HBV-endemic regions such as Eastern and Mediterranean Europe, sub-Saharan Africa (SSA), and Central, Eastern and Northern Asia [3,4]. HDV/HBV co-infection or superinfection is the most severe form of chronic hepatitis. HDV co-infection is reported to significantly suppress HBV replication [5], while other studies have reported fluctuating or persistently high levels of HBV viral loads among HDV-positive patients [6,7]. HBV/HDV infection is associated with worse clinical outcomes among people living with human immunodeficiency virus (HIV)(PLWH), resulting in a higher incidence of cirrhosis, hepatocellular carcinoma (HCC), hepatic flares, decompensation, and increased mortality [8,9]. In the largest and most recent HDV study to date in Botswana, we reported an HDV prevalence of 7.1% among PLWH who were co-infected with HBV.

The hepatitis B surface gene encodes three viral proteins, namely the large hepatitis B surface antigen [(L-HBsAg) (PreS1, PreS2 and S)], middle-HBsAg (PreS2 and S) and the small-HBsAg (S) [10]. The HBV deoxyribonucleic acid (DNA) polymerase lacks proof-reading capabilities, leading to the development of mutations which drive the evolution of the HBsAg [11,12]. The host recognizes the HBsAg through the ‘a’ determinant region that is located between amino acids 124 and 147 of the HBsAg. This induces a protective humoral immune response [11,13,14]. Therefore, mutations in this region cause changes that have an impact on HBsAg antigenicity, resulting in the development of immune-escape mutations that negatively affect the clearance of HBV infection [15,16]. Interactions not only with HDV but also with other viruses can lead to an exertion of pressure on HBV, leading to the development of mutations. The presence of other viruses and the host’s immune response may act as cofactors that modulate the genetic variability and evolution of HBV [17]. A study on the impact of HIV on HBV mutations reported the presence of fewer BCP mutations (*A1762T* and *G1764A*) coupled with more PreS2 deletions among people with HIV/HBV co-infection than those with HBV mono-infection [18]. Meanwhile, co-infection with hepatitis C virus (HCV) was associated with a higher prevalence of *A1762T*/*G1764A* mutation and a lower prevalence of the *A1752T/G*, *C1799G* and *G1896A* mutations in the precore/core promoter region of people with HBV genotype C compared to genotype B [19]. It is very important to assess the potential impact of other co-infections on the detection of HBV mutations as they might have an impact on HBV pathogenesis. These studies suggest that the volume of HBV mutations may be virus- and genotype-specific; therefore, we need to curate data on the impact of HDV on the development of HBV mutations in the most prevalent genotype (A1) in Botswana.

The World Health Organization (WHO) has recommended that all PLWH should be tested for the presence of HBV prior to initiation of combination antiretroviral therapy (cART) and those with HBV should be initiated on approved cART with anti-HBV activity such as tenofovir, emtricitabine and entecavir [20]. Implementation of these guidelines in resource-limited countries such as Botswana remains a challenge partly due to a lack of a national hepatitis treatment program despite an advanced national HIV treatment program [21,22]. Poor adherence to these drugs can lead to the emergence of HBV drug resistance mutations (DRMs), resulting in limited HBV treatment options [23]. Therefore, the impact of HDV infection on the development of unique HBV mutations among PLWH remains poorly understood in Africa, including Botswana, where HBV and HDV are not routinely tested for. This study aimed to elucidate the potential impact of HDV infection on the development of HBV mutations among PLWH in Botswana.

## 2. Materials and Methods

### 2.1. Study Participants

This was a retrospective cross-sectional study utilizing HBV whole-genome sequences (*n* = 55) generated from participants of the Botswana Combination Prevention Project (BCPP) “Ya Tsie” trial (2013–2018). To date, BCPP remains the largest pair-matched cluster-randomized trial in Botswana which enrolled a total of 12,610 participants, among which 3596 were PLWH aged between 16 and 64 years from 30 communities in Botswana [24]. Half of the BCPP communities received the standard of care, while the remaining half of the communities received HIV prevention and treatment interventions [24].

The participants’ HIV testing history was collected during all visitations in the parent study and those without documented HIV-positive status were screened using HIV rapid tests (KHB, Shanghai Kehua Bio-Engineering, Shanghai, China, and Unigold, Trinity Biotech, Bray, Wicklow, Ireland). The HIV viral load was quantified in PLWH at baseline and final visits (Abbott RealTime HIV-1 Assay, Wiesbaden, Germany) and viral suppression was defined as an HIV-1 viral load of ≤400 copies/mL. Documentation of initiation of ART (e.g., prescriptions or pills, clinical notes) was required for classifying a participant as on ART, and a CD4 cell count (Pima, Alere, Waltham, MA, USA) was performed on PLWH who were not on ART [24].

### 2.2. HBV and HDV Screening

Participants chosen for this study were PLWH who had previously been screened for HBV markers using different enzyme-linked immunosorbent assays (ELISAs). These include HBsAg (Murex HBsAg Version 2 kit, Diasorin, Dartford, UK), hepatitis B e antigen (HBeAg) (Monolisa HBe Ag/Ab, Bio-Rad, Marnes-la-Coquette, France), total hepatitis B core antibodies (anti-HBc) (Monolisa anti-HBc PLUS kit, Bio-Rad, Marnes-la-Coquette, France) and hepatitis B core antibody immunoglobulin M (anti-HBc IgM) (Anti-HBc Plus 1 Plaque, Bio-Rad, Marnes-la-Coquette, France) according to the manufacturer’s instructions [25]. The HBV viral load was quantified among HBsAg-positive participants using the COBAS AmpliPrep/COBAS TaqMan HBV Test version 2.0 (Roche Diagnostics, Mannheim, Germany), according to the manufacturer’s instructions with a detection range from 20 to 1.7 × 10^8^ IU/ mL. We screened residual samples from the parent study (stored at −80 °C) for anti-HDV antibodies at least once using the General Biologicals HDV Ab kit (General Biologicals Corporation, Taiwan, China), as per the manufacturer’s instructions. The Altona Diagnostic RealStar^®^ HDV RT-PCR 1.0 detection kit was used to determine the HDV RNA load, with slight modifications in the manufacturer’s instructions as previously described [26].

### 2.3. HBV Sequence Diversity and Mutational Analysis

We used HBV full-genome sequences that were generated using the Oxford Nanopore sequencing technology in a previous study [27]. Briefly, DNA extraction was performed on HBV-positive plasma samples using the QIAamp DNA Blood Mini kit (Qiagen, Hilden, Germany), following the manufacturer’s instructions. This was followed by PCR of the HBV whole genome on the extracted DNA samples using the HBV tilling primer pool. Library preparation of the HBV whole-genome PCR amplicons was performed with the rapid barcoding and midnight expansion protocol (version MRT_9127_v110_revH_14 Jul 2021), using HBV primers. The GridION platform (Oxford Nanopore Technologies, Oxford, UK) was used to sequence the prepared HBV DNA library with version R9.4.1 flow cells (Oxford Nanopore Technologies, Oxford, UK).

**Figure 1:**
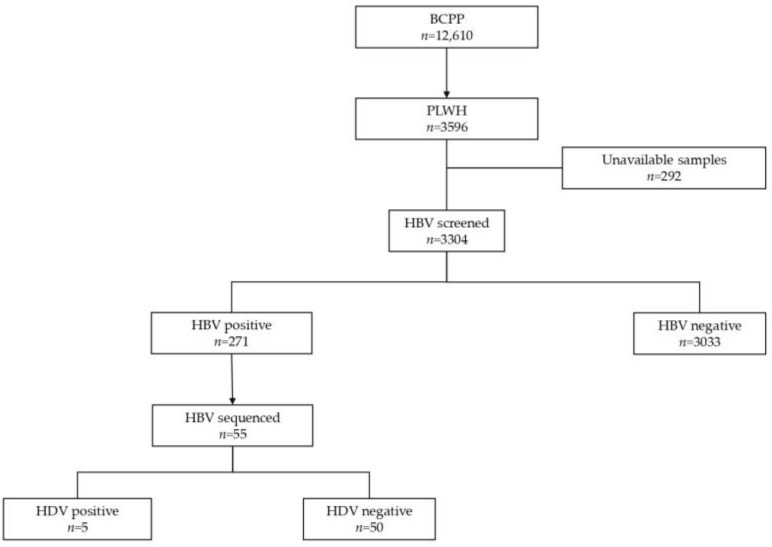
Summary flow chart of samples tested in the study.

The generated HBV sequences were aligned using AliView version 1.26 [28]. Geno2pheno [29] was used to determine HBV subgenotypes and mutations in the HBV surface region. The three domains of HBV surface proteins were manually extracted from an overlapping Pol/S fragment using the AliView version 1.26, and the impact of mutations in the S region was assessed using Phyre2 software [30]. The case group (HIV/HBV/HDV-positive) comprised participants with successfully generated HBV sequences (*n* = 5), all of whom were infected with HBV subgenotype A1. Only HBV subgenotype A1 sequences (*n* = 50) from the control group (HIV/HBV positive) were selected for diversity and mutational analysis (Figure 1).

## 3. Results

### 3.1. Participants Demographics

From participants who were HBV-positive (*n* = 271), only those with successfully generated HBV sequences (*n* = 55) were selected for this study. These participants were stratified into HIV/HBV-positive (*n* = 50) and HIV/HBV/HDV-positive (*n* = 5) groups, as shown in Table 1. HIV/HBV/HDV-positive participants, all of whom were infected with HDV clade 8, tested negative for HBeAg and anti-HBc IgM. All HIV/HBV/HDV-positive participants had an HBV viral load <2000 IU/mL compared to 72% (36/50) of HIV/HBV-positive participants.

### 3.2. Mutational Frequency and Potential Effect

HBsAg mutational analysis revealed a total of 615 mutations from both groups. Of these, there were 403 unique mutations from participants who were HIV/HBV-positive, 30 unique mutations were from HIV/HBV/HDV (*L95W*, *V96D*, *P105H*, *C138A*, *W156Q*, *K160N*, *Y161F*, *F183S*, *V184G*)-positive participants, and 182 mutations were common between the two groups (*F8L*, *T57I*, *T114S*, *E164D*, *V180A*, *A194V*, *I195M*, *S207N* and *P214L*). Among the common mutations, *S207N* (36.2%), *A194V* (34.6%) and *I195M* (19.8%) had the highest frequency.

Our findings also showed a high prevalence of mutations *S207N* (10.6%), *A194V* (10.1%), *I195M* (5.9%*)*, *E164D* (2.8%) and *K122R* (1.4%) among HIV/HBV-positive participants, with none of them exhibiting any deleterious effect. Mutations that exhibited a deleterious effect from this group include *W199R*, *C221A*, *C221S* and *W223G*. Mutations with the highest prevalence in the HIV/HBV/HDV-positive group include *S207N* (11.8%), *A194V* (11.8%) and *I195M* (5.9%), albeit with non-deleterious effects. Only mutations *L95W*, *W156Q* and *C221Y* were found to have deleterious effects in this group. All these mutations occurred in one participant whose HBV viral load was 409 IU/mL and who was receiving efavirenz, emtricitabine and tenofovir as HIV antiretroviral therapy (Table 2).

Only one mutation (*C138A*) was recorded in the ‘a’ determinant region of HBV sequences from individuals with HIV/HBV/HDV, while three mutations were recorded in HBV sequences from individuals with HIV/HDV. Additionally, five (5) mutations were recorded in the HBsAg C terminus region (aa204–206) of HBV sequences from people with HIV/HBV, while none were recorded in the HIV/HBV/HDV cohort.

## 4. Discussion

This is the first study to report on the impact of HDV infection on the development of unique HBV mutations among PLWH in Botswana. We had a total of 55 participants with HBV subgenotype A1. This is not surprising because HBV subgenotype A1 is the most prevalent strain that is circulating in Botswana [31]. None of the HIV/HBV/HDV-positive participants were positive for HBeAg or IgM anti-HBc-positive serology markers, respectively. Additionally, none of the HIV/HBV/HDV-positive participants had an HBV viral load ≥2000 IU/mL compared to 28% (14/50) of HIV/HBV-positive participants. These results are surprising because according to the literature, HIV/HBV/HDV infection is associated with severe hepatitis, which is signified by elevated HBV viral load, resulting in rapid progression to liver-related complications and mortality compared to HIV/HBV infection [8,9]. Alternatively, the low HBV viral load among participants with HIV/HBV/HDV could be attributed to the high HDV viral load coupled with the low HBV viral load observed in 60% of those participants as HDV tends to be the more dominant virus.

Several unique mutations recorded in the HIV/HBV/HDV cohort such as *L95W* and *Y161F* have previously been reported in chronic HBV liver disease patients with unknown HIV and HDV status [32]. However, this study did not elucidate their potential clinical significance on chronic HBV progression to hepatocellular carcinoma. The fact that these two mutations have previously been detected in a study without documented HDV positivity makes it difficult for us to attribute their presence or frequency to HDV positivity in our study. Substitutions in the ‘a’ determinant region of the major hydrophilic loop may lead to defective antigenicity and detection of HBsAg in the process compromising diagnosis and protection against HBV infection [33]. We report low genetic variability in the ‘a’ determinant region (located between amino acids 124 and 147) of HIV/HBV/HDV-positive participants, with only the *C138A* mutation, which was a polymorphism, seen in this region. The disulfide bonds of the cysteine residues in the antigenic loop (AGL) are critical for both the structure of the “a” determinant and reduction in HDV infectivity. The inhibition of the disulfide exchange reactions is associated with reduced HDV infectivity, which favors HBV dominance over HDV [34]. Cysteine substitutions in the AGL, including the *C138A* mutation, have previously been associated with decreased antigenicity and severely limit the binding of HBV to B- or T-cell receptors during cell-mediated immune responses [34]. This makes it harder for the host to successfully eliminate HBV, leading to the development of chronic HBV infections. We could not find any literature reports documenting the other mutations found in the HIV/HBV/HDV cohort, so they could be unique to this cohort and their potential clinical significance remains to be determined.

However, cysteine was substituted with alanine, leading to proteasomal damage in this position (Table 1). A previous study reported an increased rate of degradation after the substitution of cysteine with lysine residues in the HBV envelope protein [35]; therefore, proteasomal degradation due to alanine is a rare occurrence that needs to be studied further.

Mutations found in the ‘a’ determinant region of HIV/HBV-positive participants include *N131T*, *C139R* and *T140I* (immune escape), all of which were polymorphisms that have not been associated with proteasomal degradation in these positions. Mutations in the HBsAg C terminus region (located in amino acids 204 and 206) are detrimental to HDV replication [36]. However, in our study, position 204 was conserved, while mutations were found in position 206 (substitution of histidine and arginine in place of tyrosine) in the HIV/HBV-positive group but none were found in the HIV/HBV/HDV cohort. Deletions at positions 179 to 183, 194 to 198 and 199 to 203 of HBsAg are primarily associated with inhibition of HDV assembly [37]. Furthermore, these sites are reported to be crucial for the amplification of HBV mutations that counteract the inhibitory effects of HDV on HBV replication. No deletions were observed in our study but several mutations from both groups were found in this region. We also identified the *W199R* deleterious mutation (arginine instead of tryptophan) in the HIV/HBV-positive group, which resulted in proteasomal degradation, as well as the *P203L* mutation. Other important mutations linked with immune escape that were found outside the ‘a’ determinant region include the *K122R* mutation, which was detected among HIV/HBV-positive participants, and the *S207N* mutation, which was detected with a high frequency in both cohorts.

This study was limited by having few HDV/HBV/HIV-positive participants, which hindered comparisons using a larger cohort. The other limitation is that all participants had HBV subgenotype A1 and HDV clade 8; therefore, our findings might not necessarily be applicable to other HBV and HDV genotypes. We did not have clinical data of study participants such as fibrosis, liver enzyme levels and liver disease staging data to elucidate how they are potentially affected by HBV mutations.

## 5. Conclusions

In conclusion, our results demonstrate the presence of unique HBV mutations among the HIV/HBV/HDV-positive cohort. However, we need to study these mutations in a cohort with only HBV/HDV infection to confirm that they are solely associated with HDV infection. Interestingly, we report the presence of the *C138A* mutation which is associated with low HBV antigenicity and reduced HDV infectivity. Functional characterization of the other HBV mutations identified is recommended to determine their effect on HDV.

## Figures and Tables

**Table 1:** Demographics and clinical characteristics.

Characteristic	HIV/HBV Positive (*n* = 50)	HIV/HBV/HDV Positive (*n* = 5)
Male *n* (%)	21 (42)	2 (40)
Median Age [IQR]	42 [36–48]	46 [41–46]
HIV viral load *n* (%)		
Suppressed	47 (94)	5 (100)
Not suppressed	2 (4)	0
Unspecified	1 (2)	0
ART Status *n* (%)		
On ART	48 (96)	5 (100)
Naïve	1 (2)	0
Unspecified	1 (2)	0
ART regimen *n* (%)		
TDF-containing regimen	21 (42)	4 (80)
3TC-containing regimen	15 (30)	1 (20)
Unspecified	14 (28)	0
ARV duration in years, median (IQR)	7.9 [4.2–10.2]	5.2 [5.1–5.5]
HBV viral load *n* (%)		
Target not detected	12 (24)	1 (20)
<2000 IU/mL	24 (48)	4 (80)
≥2000 IU/mL	14 (28)	0
HBeAg positivity, *n* (%)	9 (18)	0
Anti-HBc positivity, *n* (%)	43 (86)	5 (100)
IgM anti-HBc positivity, *n* (%)	3 (6)	0
Marital status, *n* (%)		
Single or never married	35 (70)	4 (80)
Married, divorced, separated or widowed	14 (28)	1 (20)
Unspecified	1 (2)	0
Education, *n* (%)		
Non-formal and primary	24 (48)	3 (60)
Junior secondary education or higher	25 (50)	2 (40)
Unspecified	1 (2)	0

HDV—hepatitis D virus; HIV—human immunodeficiency virus; IQR—interquartile range; HBV—hepatitis B virus; HBeAg—hepatitis B e antigen; IgM anti-HBc—immunoglobulin M antibody to hepatitis B core antigen; Anti-HBc—hepatitis B core antibody; ARV—antiretrovirals; ART—antiretroviral therapy.

**Table 2:** Amino acid substitutions and their potential effect.

Wild-Type Amino Acid	Amino acid Substitutions (HIV/HBV/ HDV+)	Mutational Sensitivity	Effect	Amino acid Substitutions (HIV/HBV+)	Mutational Sensitivity	Effect
N3	-	-	-	G(2%), S(4%)	Low	Polymorphism
G7	-	-	-	G(2%)	Low	Polymorphism
F8	L(20%)	Low	Polymorphism	L(2%)	Low	Polymorphism
R24	-	-	-	K(4%)	Medium	Polymorphism
L26	-	-	-	P(2%)	Medium	Polymorphism
S45	-	-	-	T(2%), A(8%)	Low	Polymorphism
P46	-	-	-	T(2%)	Low	Polymorphism
P56	-	-	-	L(2%)	Low	Polymorphism
T57	I(20%)	Low	Polymorphism	I(6%)	Low	Polymorphism
P62	-	-	-	S(2%)	Low	Polymorphism
L95	W(20%)	High	Deleterious	-	-	-
V96	D(20%)	Medium	Polymorphism	A(11%)	Medium	Polymorphism
Q101	-	-	-	K(2%)	Low	Polymorphism
P105	H(20%)	Low	Polymorphism	-	-	-
T114	S(20%)	Low	Polymorphism	S(4%)	Low	Polymorphism
K122	-	-	-	R(15%)	Low	Polymorphism
N131	-	-	-	T(2%)	Low	Polymorphism
C138	A(20%)	Low	Polymorphism	-	-	-
C139	-	-	-	R(2%)	Medium	Polymorphism
T140	-	-	-	I(2%)	Low	Polymorphism
W156	Q(20%)	High	Deleterious	-	-	-
A159	-	-	-	I(2%)		Polymorphism
K160	N(20%)	Low	Polymorphism	-	-	-
Y161	F(20%)	Low	Polymorphism	-	-	-
E164	D(60%)	Low	Polymorphism	D(30%)	Low	Polymorphism
A166	-	-	-	D(2%)	Medium	Polymorphism
V180	A(20%)	Low	Polymorphism	A(8%)	Low	Polymorphism
F183	S(20%)	Medium	Polymorphism	-	-	-
V184	G(20%)	Low	Polymorphism	W(2%), A(2%), R(2%)	Low	Polymorphism
G185	-	-	-	W(2%)	Low	Polymorphism
P188	L(20%)	Medium	Polymorphism	L(2%)	Low	Polymorphism
V190	-	-	-	I(2%)	Medium	Polymorphism
V194	-	-	-	A(2%)	Low	Polymorphism
I195	M(60%)	Low	Polymorphism	M(55%)	Low	Polymorphism
M198	-	-	-	V(2%)	Low	Polymorphism
W199	-	-	-	R(4%)	High	Deleterious
P203	-	-	-	L(2%), Q(4%)	Medium	Polymorphism
L205	-	-	-	Q(2%), C(2%), S(2%)	Medium	Polymorphism
Y206	-	-	-	H(4%), R(6%)	Medium	Polymorphism
S207	N(12%)	Medium	Polymorphism	N(2%)	Medium	Polymorphism
I213	-	-	-	M 2%)	Medium	Polymorphism
P214	-	-	-	L(4%)	Medium	Polymorphism
L215	-	-	-	P(2%)	Medium	Polymorphism
C221	Y(20%)	High	Deleterious	A(2%)	High	Deleterious
W223	-	-	-	G(2%)	High	Deleterious

HDV—hepatitis D virus; HIV—human immunodeficiency virus; HBV—hepatitis B virus; A—alanine; C—cysteine; D—aspartic acid; E—glutamic acid; F—phenylalanine; G—glycine; H—histidine; I—isoleucine; K—lysine; L—leucine; M—methionine; N—asparagine; P—proline; Q—glutamine; R—arginine; S—serine; T—threonine; W—tryptophan; Y—tyrosine; V—valine.

## Data Availability

The data presented in this study are available upon request from the corresponding author. The data are not publicly available as the sequences are currently being analyzed for other objectives of the bigger project.
